# Penetrating injuries in Germany – epidemiology, management and outcome an analysis based on the TraumaRegister DGU®

**DOI:** 10.1186/s13049-021-00895-1

**Published:** 2021-06-13

**Authors:** D Bieler, E Kollig, L Hackenberg, JH Rathjen, R Lefering, A Franke, the Committee on Emergency Medicine, Intensive Care and Trauma Management(Sektion NIS) of the German Trauma Society (DGU)

**Affiliations:** 1 Department of Orthopaedics and Trauma Surgery, Reconstructive Surgery, Hand Surgery, Plastic Surgery and Burn Medicine, German Armed Forces Central Hospital, Koblenz, Germany; 2 Department of Orthopaedics and Trauma Surgery, Medical Faculty and University Hospital Düsseldorf, Heinrich-Heine-University, Moorenstraße 5, 40225 Düsseldorf, Germany; 3 Institute for Research in Operative Medicine, Witten/Herdecke University, Cologne, Germany

**Keywords:** gunshvot wound, stab wound, TraumaRegister DGU®, penetrating injuries, Germany, epidemiology

## Abstract

**Background:**

The management of penetrating wounds is a rare challenge for trauma surgeons in Germany and Central Europe as a result of the low incidence of this type of trauma. In Germany, penetrating injuries are reported to occur in 4–5 % of the severely injured patients who are enrolled in the TraumaRegister DGU® (trauma registry of the German Trauma Society). They include gunshot injuries, knife stab injuries, which are far more common, and penetrating injuries of other origin, for example trauma caused by accidents. The objective of this study was to assess the epidemiology and outcome of penetrating injuries in Germany, with a particular focus on the level of care provided by the treating trauma centre to gain more understanding of this trauma mechanism and to anticipate the necessary steps in the initial treatment.

**Materials and methods:**

Since 2009, the TraumaRegister DGU® has been used to assess not only whether a trauma was penetrating but also whether it was caused by gunshot or stabbing. Data were taken from the standard documentation forms that participating German hospitals completed between 2009 and 2018. Excluded were patients with a maximum abbreviated injury scale (MAIS) score of 1 with a view to obtaining a realistic idea of this injury entity, which is rare in Germany.

**Results:**

From 2009 to 2018, there were 1123 patients with gunshot wounds, corresponding to a prevalence rate of 0.5 %, and 4333 patients with stab wounds (1.8 %), which were frequently caused by violent crime. The high proportion of intentionally self-inflicted gunshot wounds to the head resulted in a cumulative mortality rate of 41 % for gunshot injuries. Stab wounds were associated with a lower mortality rate (6.8 %). Every fourth to fifth patient with a gunshot or stab wound presented with haemorrhagic shock, which is a problem that is seen during both the prehospital and the inhospital phase of patient management. Of the patients with penetrating injuries, 18.3 % required transfusions. This percentage was more than two times higher than that of the basic group of patients of the TraumaRegister DGU®, which consists of patients with a MAIS ≥ 3 and patients with a MAIS of 2 who died or were treated on the intensive care unit.

**Conclusions:**

In Germany, gunshot and stab wounds have a low incidence and are mostly caused by violent crime or attempted suicides. Depending on the site of injury, they have a high mortality and are often associated with major haemorrhage. As a result of the low incidence of these types of trauma, further data and analyses are required in order to provide the basis for evaluating the long-term quality of the management of patients with stab or gunshot wounds.

## Introduction

As a result of the low incidence of gunshot and stab wounds in Germany and Central Europe, the management of these types of penetrating injuries is a rare challenge for surgeons during and after resuscitation room care. In Germany, penetrating injuries are reported to occur with an incidence of 4.1 %.[1] Apart from annual police-recorded crime statistics *(Polizeiliche Kriminalstatistik, PKS)* on the use of firearms and stab weapons in association with criminal offences, no robust data on the epidemiology of associated injuries are available in Germany. Crime statistics, too, provide only limited information on injury patterns and in particular on the severity of injuries since these data are collected on the basis of legal criteria and have not yet been processed for medical purposes.

**Fig. 1:**
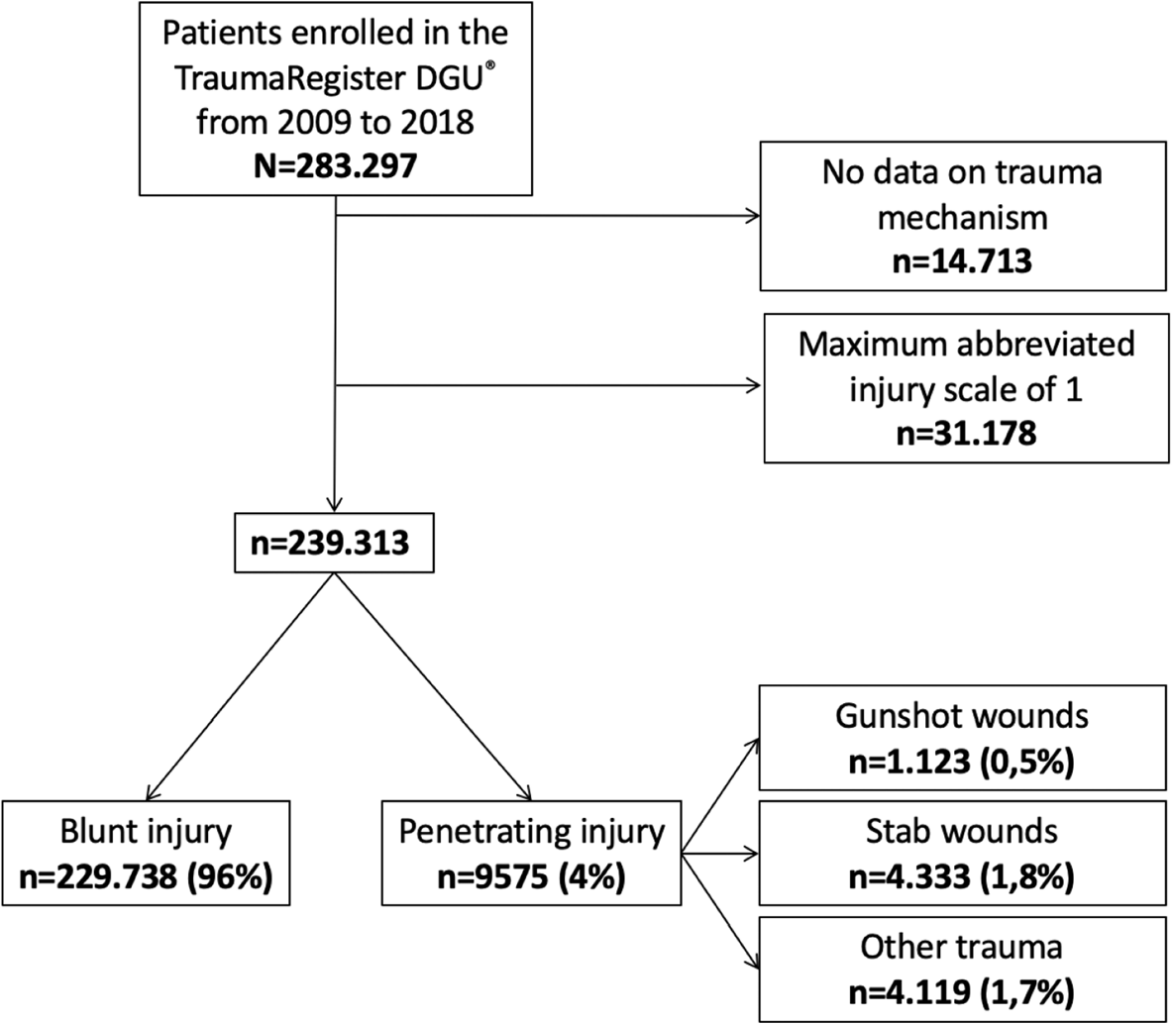
Cohort identification

According to police-recorded crime statistics from 2018, a total of 8343 incidents involving the use of firearms were reported in Germany. This figure is consistent with the downward trend of previous years. In 3819 (46 %) of the registered cases, firearms were used as a threat but not actually fired. In 54 % (*n* = 4524) of the cases, firearms were actually fired (Fig. 1).[2] These statistical data do not include the use of firearms by law enforcement officers but are limited to criminal offences.

Although a certain number of cases may have gone unreported, these figures provide a realistic idea of the incidence of gunshot wounds in Germany. Crime statistics can neither answer the question as to what clinical role and what demand for resources are associated with the management of this injury entity in civilian trauma centres, nor do they provide a precise picture of injury patterns and outcomes.

Since prospective studies on this matter are currently not available in Germany, we conducted a retrospective investigation on the basis of the TraumaRegister DGU® (trauma registry of the German Trauma Society).

The objective of this study was to assess how often patients with gunshot and stab wounds were treated in the resuscitation room and what injury patterns they presented. We not only assessed patient demographics, the location of injury (extremities or body cavities), prehospital treatment, and inhospital treatment but, where possible, also collected data on the length of hospital stay and mortality.

We know from the literature that penetrating injuries can develop in a very dynamic manner.[3] For this reason, it is important to know what criteria indicate the need for specific treatment and what resources must be held available and are required. The present study also addressed these aspects.

Such a cohort design creates a bias since the study does not include data, for example, on patients who sustained only minor gunshot injuries and were not admitted to the resuscitation room or who received initial (and definitive) treatment in a hospital that does not participate in the TraumaRegister DGU®. There is, however, currently no German database or similar registry that can provide the basis for a general and comprehensive analysis covering a period of several years.

## Materials and methods

The TraumaRegister DGU® of the German Trauma Society (Deutsche Gesellschaft für Unfallchirurgie, DGU) was founded in 1993. The aim of this multi-centre database is the pseudonymised and standardised documentation of severely injured patients.

Data are collected prospectively in four consecutive time phases from the site of the incident until discharge from hospital: (A) prehospital phase, (B) emergency/resuscitation room and initial surgery, (C) intensive care unit, and (D) discharge. Documentation includes detailed information on demographics, injury patterns, comorbidities, prehospital and inhospital management, course on intensive care unit, relevant laboratory findings including transfusion data, and outcome. Included are patients who are admitted to hospital via the resuscitation room and subsequently receive intensive or intermediate care and patients who arrive at hospital with vital signs and die before admission to the intensive care unit.

The infrastructure for documentation, data management, and data analysis is provided by the Academy for Trauma Surgery (Akademie der Unfallchirurgie GmbH, AUC), which is affiliated with the German Trauma Society. Scientific leadership is provided by the Committee on Emergency Medicine, Intensive Care and Trauma Management (Sektion NIS) of the German Trauma Society. Participating hospitals submit their pseudonymised data to a central database via a web-based application. Scientific data analysis is approved according to a peer review procedure established by Sektion NIS.

The participating hospitals are primarily located in Germany (90 %), but a growing number of hospitals in other countries contribute data as well (i.e. Austria, Belgium, China, Finland, Luxembourg, Slovenia, Switzerland, the Netherlands, and the United Arab Emirates). Currently, approximately 33,000 cases (basic group of patients) from more than 650 hospitals are entered into the database per year.

Participation in TraumaRegister DGU® is voluntary. For hospitals associated with the TraumaNetzwerk DGU®, the entry of at least a basic data set is obligatory for reasons of quality assurance. Approximately 50 % of all cases, however, are documented on the basis of the standard dataset.

Since 2009, the TraumaRegister DGU® has been used to assess not only whether a trauma was penetrating but also whether it was caused by gunshot or stabbing. These data allowed us to identify relevant cases and to define the study period. We distinguished three subgroups of injuries, i.e. gunshot wounds, stab wounds, and other penetrating injuries.

Data were taken from the standard documentation forms that participating German hospitals completed between 2009 and 2018. Excluded were patients with a maximum abbreviated injury scale (AIS) score of 1. We did not specify any other exclusion criteria in order to obtain as comprehensive a picture as possible of the injury entities investigated in this study.

As a result of the high proportion of gunshot wounds to the head (anatomical AIS body region code 1: head without face and facial bones) and the high overall mortality rates that are reported in the literature for these injuries, a subgroup of patients with head injuries was analysed as well. In addition, we analysed two-cavity injuries (thoracic and abdominal injuries) because of their clinical and therapeutical relevance. Inclusion and exclusion criteria were identical for all subgroups.

Primary endpoints were mortality and length of hospital stay. Secondary endpoints were transfusions (units of packed red blood cells), massive transfusions (more than 10 units of packed red blood cell units), fluid administration, chest drain insertion for thoracic injuries with an AIS ≥ 3. Other secondary endpoints were emergency operations, which were not registered in a standardised manner during the study period. For this reason, the analysis of emergency operations was limited to cases that were reported from 2015 to 2018 since data on different types of emergency operations have been entered into the registry only since 2015. Especially emergency thoracotomy for thoracic injuries and emergency laparotomy for abdominal injuries were defined as secondary endpoints in the analysis of cases from 2015 to 2018 since every emergency operation – and not only the first emergency surgical procedure – was registered from that time on.

SPSS® (version 24, IBM Corp., Armonk, NY, United States) was used for the analysis of descriptive statistics. Numbers of cases, percentages, means, and standard deviations (SD) were provided. Because of the large number of cases and the wide variety of possible comparisons (multiple parameters, three-group comparisons), formal statistical tests were performed only for specific variables (e.g. rescue times). The chi-squared test was used for categorical variables, and the Mann-Whitney *U* test and the Kruskal-Wallis test were used for metric variables. Missing data were handled by using pairwise case exclusion.

Differences in time of more than five minutes, in percentages of 5 % or more, and in volume of more than 250 mL were regarded as clinically relevant.

The study was performed in accordance with the publication guideline of the TraumaRegister DGU® and is registered as TR-DGU Project ID 2016-031. Since the study was a retrospective anonymised analysis, ethical approval was not required according to the regulations of the responsible regional medical association.

## Results

Between 2009 and 2018, there were 9575 patients with a penetrating injury, accounting for 4.0 % of the cohort of patients with an AIS > 1 and with data on trauma mechanism.

Of these, 1123 had a gunshot injury, which corresponds to 0.5 % of the study cohort, and 4333 had a stab wound, which corresponds to 1.8 % of the cohort. These types of injury thus accounted for more than half (57 %) of the penetrating injuries that were included in the TraumaRegister DGU® (Fig. 1).

### General data

The majority of patients with penetrating injuries were male (89.3 %). The mean / median patient age was 44 / 42 years. Gunshot injuries were associated with a mean / median injury severity score (ISS) of 22.9 / 25, which was the highest overall injury severity score of all three subgroups. Patients with stab wounds had the lowest ISS on admission (mean 13.9; median 10) and patients with other penetrating trauma had a mean / median ISS of 17.5 / 14 (Table 1). According to documented diagnoses, patients with penetrating injuries rarely presented with single injuries. A median of three diagnoses was documented for patients with gunshot and stab wounds. Patients with other penetrating injuries had a median of four diagnoses. Furthermore, patients with penetrating injuries were often treated in (supraregional) level 1 trauma centres. The percentage of gunshot injuries that were managed in level 1 trauma centres was particular high (72.2 %). Patients with penetrating injuries were rarely treated in local (level 3) trauma centres, accounting for 4.9–11 % of the subgroups.

**Table 1:** General data (m = mean, SD = standard deviation, IQR = interquartile range, ASA = American Society of Anesthesiologists, ISS = injury severity score)

	Gunshot (*n* = 1123)		Stab (*n* = 4333)		Other (*n* = 4119)	
Male (n)	1001	89.3 %	3627	83.8 %	3125	76.1 %
Age (m)	52.6	SD 21	39.4	SD 17.2	46.8	SD 21.3
Age (median, IQR)	53	(36–71)	36	(26–51)	47	(28–62)
Age > 70 years (n)	306	27.3 %	301	7.0 %	1324	13.9 %
Age > 16 years (n)	11	1.0 %	66	1.5 %	186	4.5 %
Level 1 trauma care	811	72.2 %	2474	57.1 %	2414	58.6 %
Level 2 trauma care	257	22.9 %	1364	31.5 %	1226	29.8 %
Level 3 trauma care	55	4.9 %	473	10.9 %	455	11.0 %
ASA class 3–4	191	19.6 %	371	9.4 %	442	12.1 %
ISS (m)	22.9	SD 15.2	13.9	SD 10.1	17.5	SD 12.8
ISS (median, IQR)	25	(12,13,14,15,16,17,18,19,20,21,22,23,24,25,26)	10	(8,9,10,11,12,13,14,15,16,17,18)	14	(9,10,11,12,13,14,15,16,17,18,19,20,21,22)
Number of documented diagnoses (median, IQR)	3	(2,3,4,5)	3	(2,3,4)	4	(2,3,4,5,6)

### Trauma mechanism

An isolated analysis of the gunshot wounds that were included in the registry between 2009 and 2018 (*n* = 1123) and were treated in the resuscitation room showed that 55.8 % of the cases were intentionally self-inflicted injuries. By contrast, accidents involving the use of firearms accounted for only a small annual number of cases (*n* = 10). Violent crime was suspected as the cause of gunshot injuries in 34.5 % of cases.

**Fig. 2:**
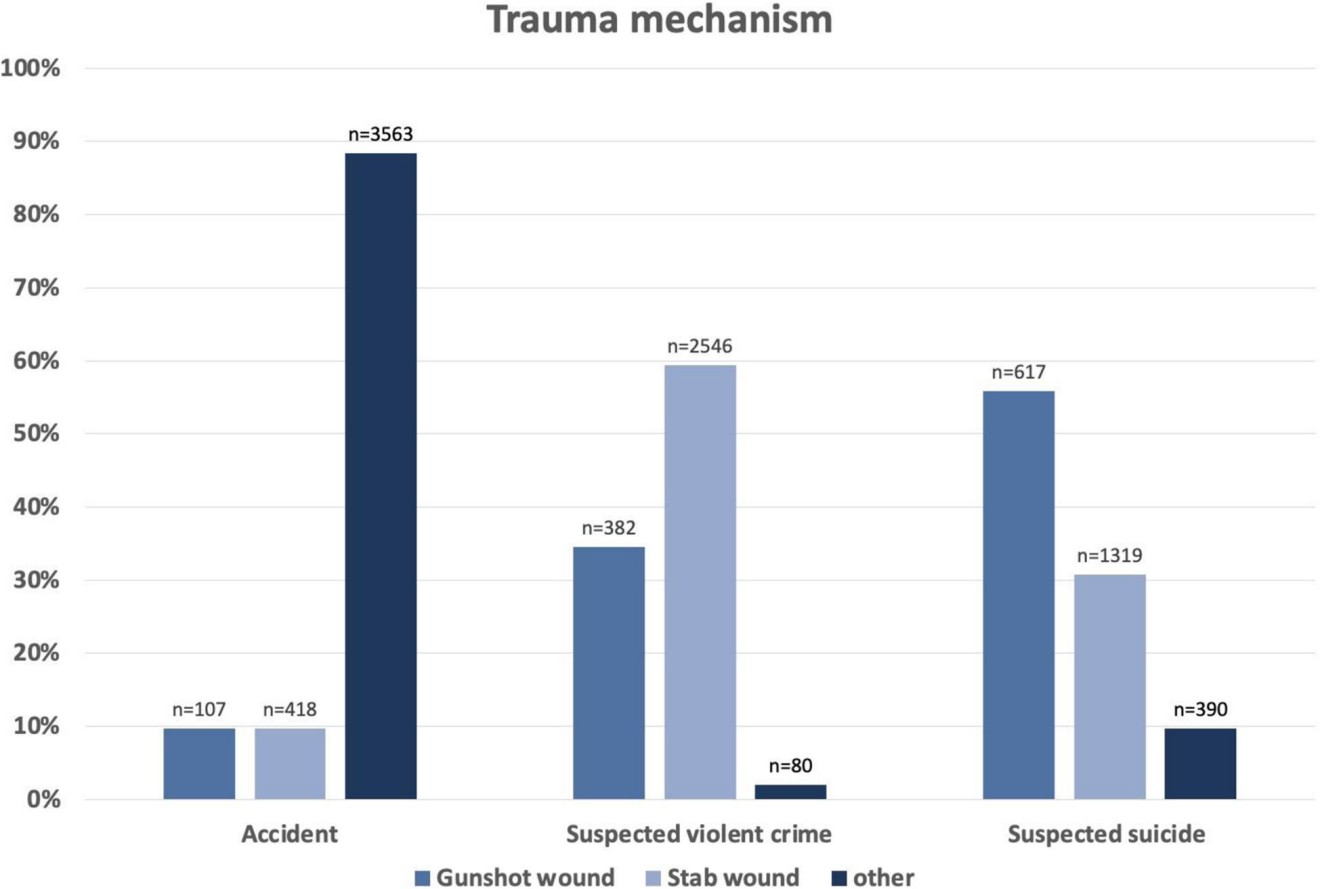
Distribution of trauma mechanisms in the three subgroups of TraumaRegister DGU® patients with penetrating injuries (*n* = 9575) (other = other mechanisms of trauma, *n* = number of cases)

Compared with the number of gunshot injuries, almost twice as many stab wounds (59.4 %) were caused by violent crime, whereas suicide attempts accounted for only 30.8 % of stab wounds that were managed in the resuscitation room. Accidents were the cause of stab wounds in only 9.8 % of cases. Other penetrating injuries were associated with a completely different distribution of injury mechanisms (Fig. 2).

**Fig. 3:**
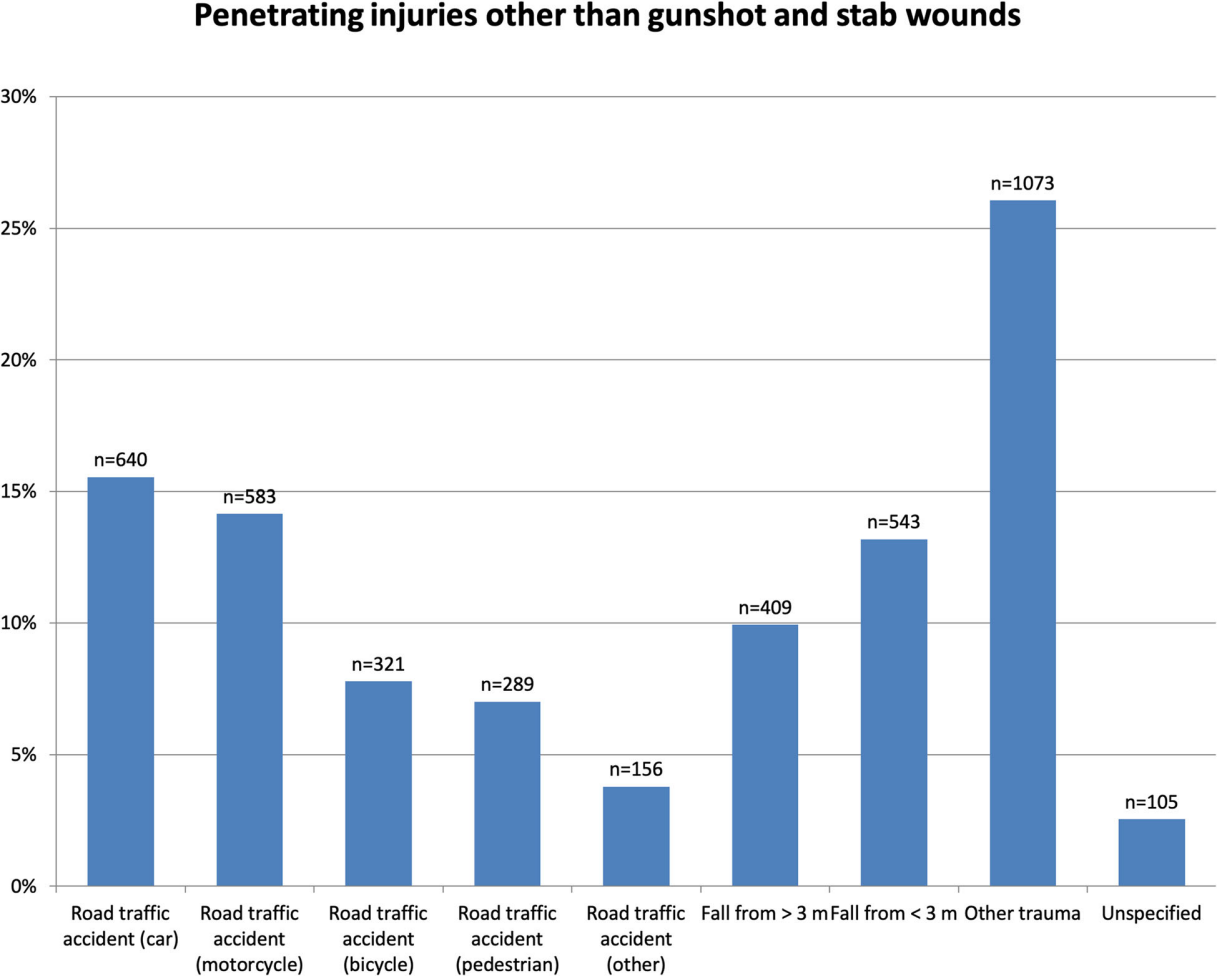
Trauma mechanisms in penetrating patients with injuries other than gunshot and stab wounds (*n* = number of cases)

Heterogeneous causes of injury were identified through an analysis of trauma mechanisms in the group of patients with penetrating injuries other than gunshot or stab wounds (Fig. 3). These injuries were most commonly caused by traffic accidents involving cars (15.5 %) and motorcycles (14.2 %) and by other trauma (26.1 %). Traffic accidents involving cyclists and pedestrians accounted for 7–8 % each and falls for approximately 23 % of other penetrating injuries.

### Patterns of injury

In the subgroup of patients with gunshot injuries, the head (52.2 %) was the most commonly affected anatomical region, followed by the chest (23.9 %) and the abdomen (20.4 %). Injuries to the extremities were documented in 19.7 % of cases with gunshot injuries.

Stab wounds most commonly involved the chest (52 %) and the abdomen (37.4 %). At least one of these two cavities was affected in 76 % of the documented cases.

Penetrating injuries other than gunshot and stab wounds involved the head (34.3 %), the chest (33.6 %), and the upper (35.6 %) and lower (41.3 %) extremities.

**Fig. 4:**
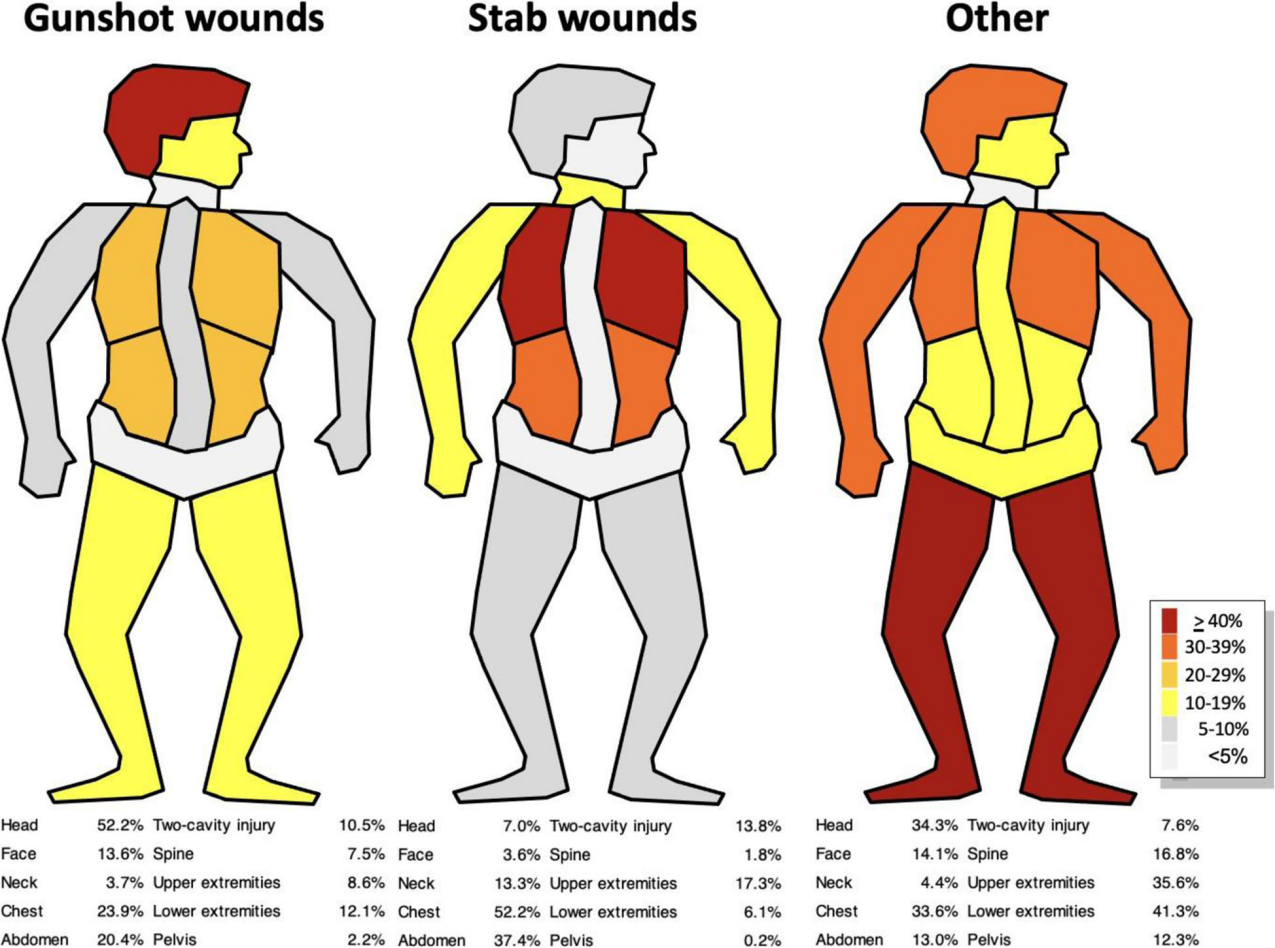
Distribution of injuries in the subgroups (gunshot, stab and other penetrating wounds) of TraumaRegister DGU® patients with penetrating injuries (*n* = 9575). One injury can involve multiple body regions. Two-cavity injuries are injuries to the chest and abdomen

Figure 4 provides a detailed overview of the distribution of injuries.

**Fig. 5:**
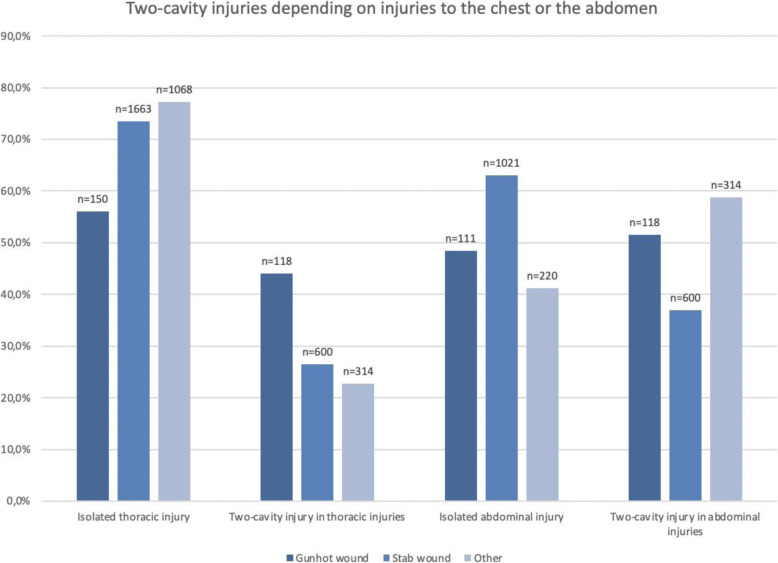
Two-cavity injuries in TraumaRegister DGU® patients with penetrating injuries depending on injuries to the chest or the abdomen (other = penetrating injuries other than gunshot and stab wounds, two-cavity injury = injury to the chest and the abdomen)

An analysis of two-cavity injuries revealed that more than a quarter of thoracic stab wounds also involved the abdomen and that almost half of the gunshot injuries to the chest (44 %) also affected the abdominal region. More than 50 % of abdominal gunshot injuries and 37 % of abdominal stab wounds also involved the chest. Other penetrating abdominal injuries also affected the chest in almost 60 % and other penetrating thoracic injuries also involved the abdomen in 23 % (Fig. 5).

Registry data from 2015 to 2018 showed that 21.4 % of the patients with thoracic trauma underwent acute thoracotomy. The majority of these patients (38.8 %) had sustained gunshot injuries. Urgent laparotomy was performed in 66.9 % of the patients with penetrating abdominal injuries, the majority of whom presented with gunshot injuries (76.5 %). The decision to perform thoracotomy or laparotomy strongly depended on injury severity. Thoracotomy was performed in 8 % and laparotomy in 53 % of the patients with an AIS score of 2. By contrast, thoracotomy was performed in as much as 41 % and laparotomy in as much as 81 % of the patients with an AIS score of 5.

### Prehospital and inhospital management

Since head injuries accounted for a significantly higher percentage of gunshot injuries compared with stab and other penetrating injuries, gunshot injuries were also associated with a significantly lower Glasgow Coma Scale (GCS) score (Table 2, *p* < 0.001) than other penetrating injuries. Furthermore, the on-scene time was considerably lower for stab injuries (21.3 min) than for gunshot injuries (29.5 min). Times from scene to hospital varied by up to three minutes. As a result, stab injuries were associated with significantly lower prehospital times than gunshot and other penetrating injuries (*p* < 0.001) (Table 2). Air transportation was less commonly used for patients with stab wounds (only approximately 6 %) than for the other patients (*p* < 0.001).

**Table 2:** Prehospital and inhospital management (*only data from standard documentation forms were used since the QM documentation form does not include relevant parameters); GCS = Glasgow Coma Scale, ED = emergency department, RISC = Revised Injury Severity Classification, SMR = standardised mortality rate, m = mean, SD = standard deviation, PRBC = packed red blood cells, CI = confidence interval

	Gunshot *n* = 1123		Stab *n* = 4333		Others *n* = 4119		
***Prehospital data***							
Prehospital GCS score (m +/-)	9.7	SD 5.4	13.4	SD 3.3		12.6	SD 4
Prehospital shock (n)	177	20.7 %	863	24.3 %		572	16.7 %
On-scene time (m +/-)* in minutes	29.5	SD 17.7	21.3	SD 13.9		28.4	SD 16.7
Prehospital time (m +/-) in minutes	67.6	SD 44.2	58	SD 46.2		64.1	SD 35.6
Air transportation (n)	203	20.6 %	227	5.7 %		893	23.7 %
***Prehospital management***							
Prehospital fluid administration (n)	827	87.2 %	3415	87.4 %		3319	89.5 %
Prehospital fluid administration in mL (m +/-)	875	SD 681	856	SD 695		919	SD 729
Prehospital intubation (n)	521	51.9 %	715	17.7 %		1177	30.7 %
Prehospital chest drain insertion (n)*	28	5.0 %	96	5.4 %		53	3.0 %
Prehospital catecholamine therapy (n)*	107	19.1 %	159	9.0 %		183	10.5 %
Resuscitation (n)	72	7.2 %	181	4.5 %		135	3.5 %
Prehospital sedation (n)*	387	69.2 %	876	49.4 %		1235	70.6 %
***Clinical management***							
Shock on arrival at ED (n)	182	17.8 %	684	17.1 %		493	13.4 %
Fluid administration at ED in mL (m +/-)*	1286	SD 1632	1460	SD 1602		1318	SD 1473
PRBC transfusions (n)	188	17.1 %	834	19.4 %		664	16.3 %
Massive transfusions (> 10 PRBC units, n)	31	2.8 %	139	3.2 %		115	2.8 %
Emergency operations (since 2015)	184	46 %	916	48 %		484	43 %
Emergency laparotomy	68	18 %	568	31 %		59	6 %
Emergency thoracotomy	33	9 %	240	13 %		30	3 %
Length of hospital stay (in days)	11.8	SD 16.3	9	SD 10.7		18.5	SD 22.5
Died within 6 h	156	13.9 %	158	3.6 %		177	4.3 %
Died (n)	429	38.2 %	289	6.7 %		455	11.0 %
RISC II score (%)		35.7 %		8.6 %			13.2 %
SMR (with 95 % CI)	1.07	0.99–1.15	0.78	0.69–0.86		0.84	0.76–0.91

Since patients with gunshot injuries had a lower GCS score, intubation was significantly more common in this patient group during prehospital management. Patients with gunshot injuries also more often required other types of prehospital treatment such as resuscitation, catecholamine therapy, and sedation.

**Fig. 6:**
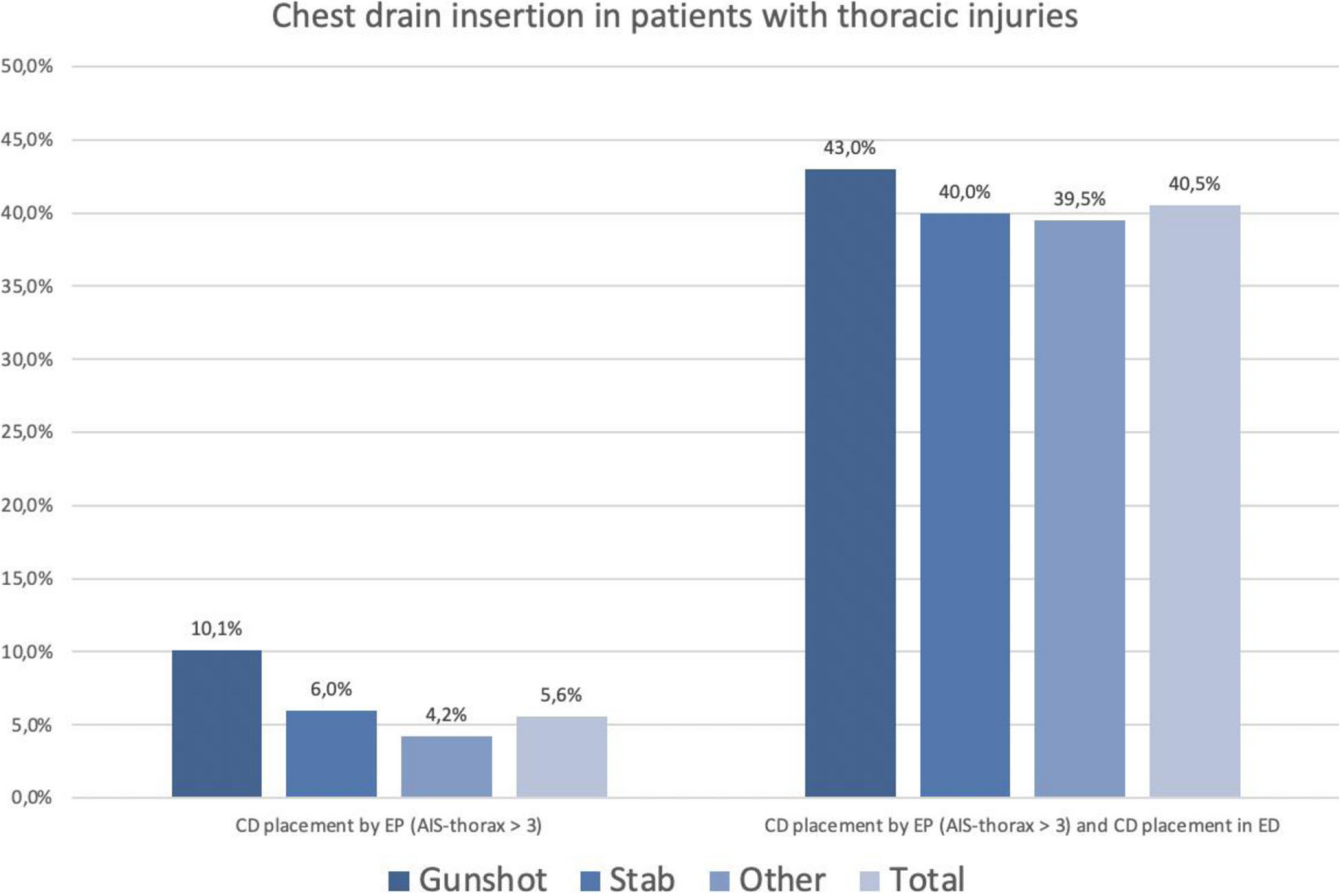
Chest drain insertion in patients with penetrating injuries to the chest (AIS≥3) on the basis of TraumaRegister DGU® data; CD = chest drain, EP = emergency physician, ED = Emergency department

Patients with penetrating injuries required chest drain insertion only rarely (approximately 5 %). Chest drains were placed in only about every tenth patient with a penetrating gunshot injury to the chest and in about every twentieth patient with a stab or other penetrating injury to the chest. Figure 6 shows how often the different types of penetrating thoracic injuries (AIS ≥ 3) mandated chest drain insertion in the prehospital setting and in the resuscitation room.

Apart from the small number of cases in which chest drains were placed during the prehospital phase, it is interesting to note that almost every second patient who underwent prehospital chest drain insertion required an additional chest drain or chest drain replacement in the resuscitation room.

**Fig. 7:**
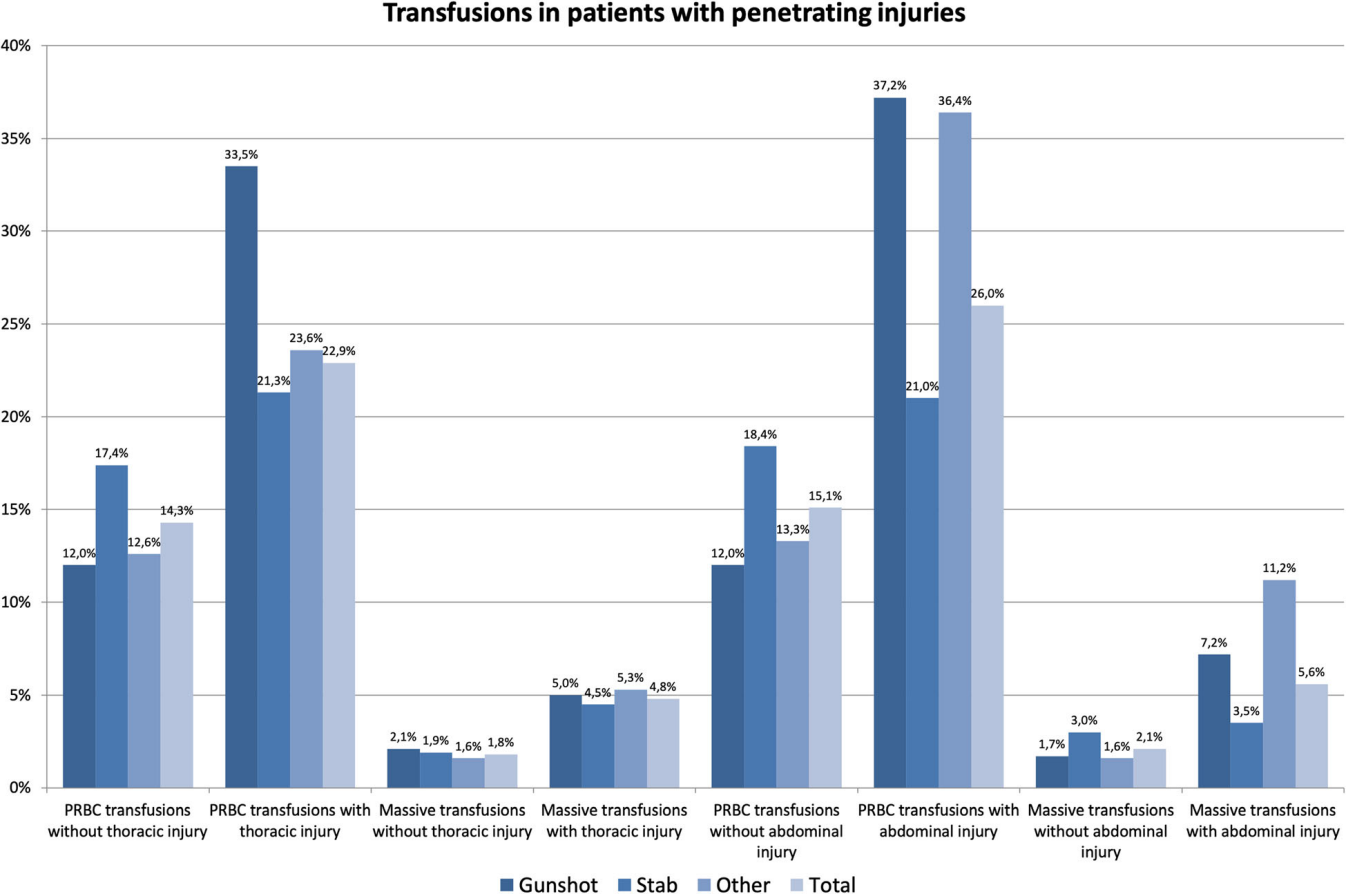
Transfusion requirements in patients with penetrating injuries depending on injury patterns on the basis of TraumaRegister DGU® data (pRBC = packed red blood cells)

An analysis of (massive) transfusion requirements for penetrating injuries within the first 48 h of admission shows heterogeneous results (Fig. 7). The pattern of injury had a significant effect on transfusion rates especially in patients with thoracic or abdominal injuries. The presence of thoracic or abdominal injuries was associated with up to three times higher transfusion rates. Massive transfusions too were required up to three times more often in patients with thoracic or abdominal injuries.

### Outcome

**Fig. 8:**
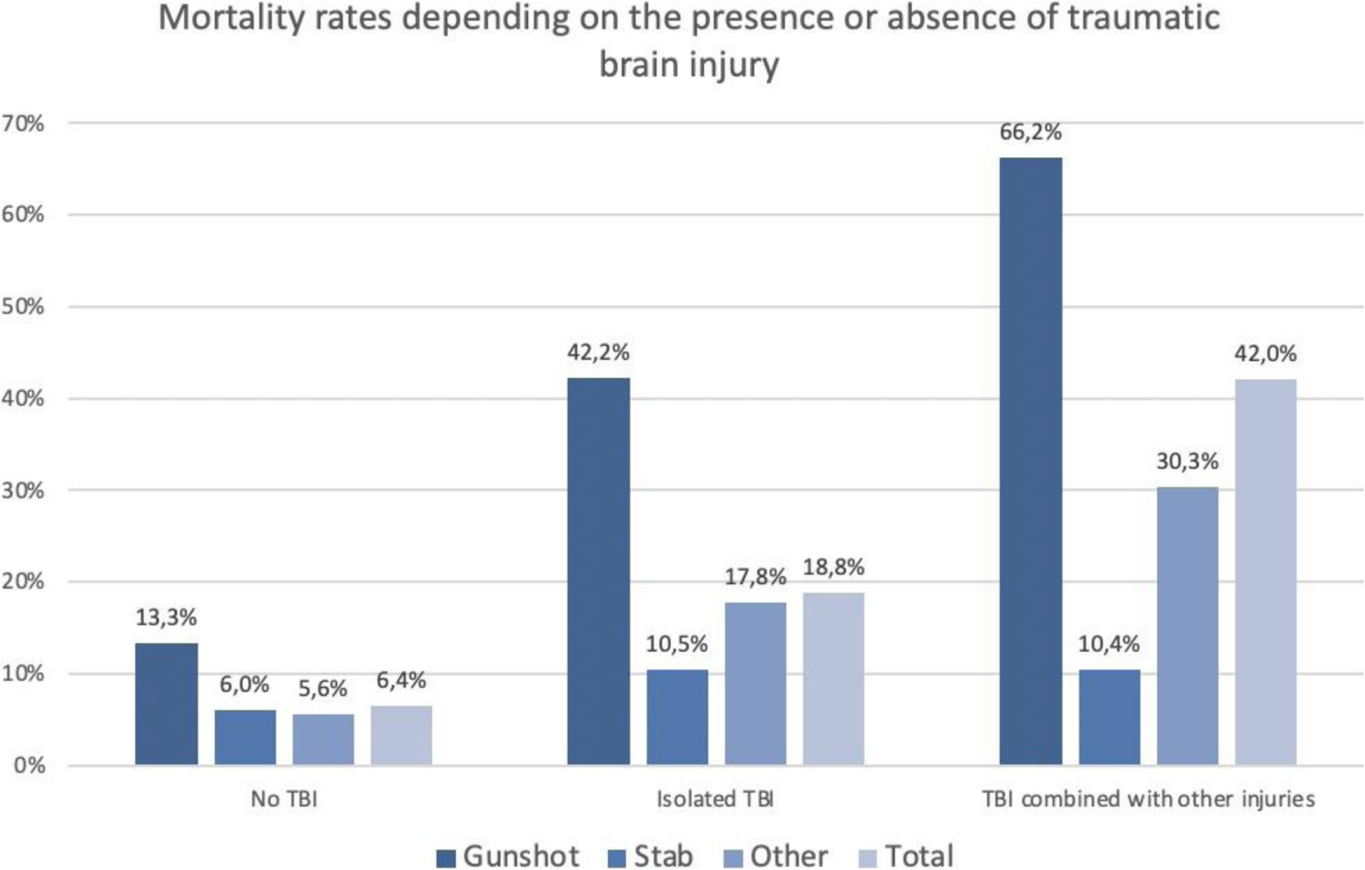
Mortality rates for patients with penetrating injuries depending on the presence or absence of traumatic brain injury (TBI) on the basis of TraumaRegister DGU® data

As a result of the high proportion of suicide-related gunshot wounds to the head, the mean mortality rate for gunshot injuries was high (38.2 %) whereas the mortality rate for gunshot injuries that did not involve the head was considerably lower (13.3 %). Different results were obtained for the mortality rates in the other two subgroups (Table 2). Figure 8 shows the mortality rates for the different subgroups of penetrating injuries depending on the presence or absence of a traumatic brain injury. In the group of patients with gunshot injuries, 52.2 % of those who died had a traumatic brain injury (TBI), either in isolation or in combination with other injuries. In the group of patients with stab wounds, 93 % of those who died had no TBI. Revised Injury Severity Classification (RISC) II prognostic scores and standardised mortality rates (SMR) for the three groups of patients are shown in Table 2. Based on RISC II scores, gunshot injuries were associated with an increased SMR (1.07) whereas stab wounds (0.78) and other penetrating injuries (0.84) were associated with lower SMRs.

## Discussion

This study is the first to provide an overview of penetrating injuries in Germany over a period of ten years. It is based on patients from the TraumaRegister DGU® who met the criterion of having sustained potentially life-threatening injuries.

Registry data show that half of all penetrating injuries (4 %) are caused by gunshot or stabbing. This injury entity is thus considerably rarer in Germany than in the United States (20 %).[4]

Although a medical registry cannot replace crime statistics, it shows that the causes of perforating injuries are suspected suicide or violent crime in the majority of cases. Accidents involving the use of firearms or stabbing weapons are far less commonly documented. The incidence of these accidents is comparable to that reported in the United States.[4]

The total number of patients with penetrating injuries that were treated in resuscitation rooms in Germany was 9575 during the period from 2009 to 2018, corresponding to a mean annual number of 957 patients.

A decreased level of consciousness was documented as a leading life-threatening condition, especially in patients with a head injury caused by the use of firearms in suspected suicide attempts. Although these patients are managed by intubation and intensive care, their overall mortality rate is three to five times higher than that reported for patients without a gunshot injury to the head. It is interesting to note that gunshot injuries to the head are isolated injuries in approximately 50 % of the cases. This percentage is similar to results from other studies, which too report mortality rates of more than 40 % [5–7]. These patients usually require no intervention other than airway and circulatory procedures. The management of these patients focuses on deciding whether, depending on injury severity, emergency surgery or further diagnostic and imaging procedures should be performed [8].

Penetrating injuries involving other regions of the body may require a more differentiated treatment approach [3].

In this study, at least a fifth of the patients presented with a penetrating injury to the extremities. Several authors reported that the use of tourniquets significantly reduced mortality from exsanguination from extremity injuries [9–11]. Although tourniquets have been primarily used by the military, experience has in recent years encouraged the use of tourniquets also in the civilian setting with a view to avoiding preventable deaths from extremity haemorrhage with prehospital tourniquet application [12]. As a result of this development, isolated extremity injuries are today associated with a low mortality rate since extremity haemorrhage can be easily and adequately controlled with tourniquets [13, 14]. In our opinion, the risks of ischaemic or neurologic complications are acceptable given the fact that tourniquets can save lives. It should be noted that potential tourniquet-related (ischaemic and pressure) damage can be expected to be fully reversible since prehospital rescue times of 68 min have been reported for gunshot wounds and 58 min for stab wounds in Germany [15, 16]. Tourniquets should nevertheless only be used for specific indications. If the situation permits, pressure dressings continue to be the treatment of choice for non-spurting wounds.

Injuries involving one of the large body cavities (thoracic or abdominal cavity) also affect the other large body cavity in up to 50 % of the cases and are referred to as two-cavity injuries. Haemodynamically stable patients (75–80 % in our cohort) undergo diagnostic computed tomography (CT) with a view to improving surgical planning. When the patient is positioned for surgery, the presence of a two-cavity injury must be assumed unless the involvement of both cavities can definitely be ruled out. Moreover, two-cavity injuries are associated with a significantly higher probability that the patient requires blood products. The results of this study are in line with those reported in the literature [17]. Emergency operations had to be performed almost twice as often in patients with penetrating injuries (46 %) than in the basic group of patients of the TraumaRegister DGU® during the ten-year study period (*n* = 242,793; emergency and early operations, 23.5 %) [1]. Emergency thoracic and abdominal operations for gunshot and stab wounds (thoracotomy, 9 and 13 %; laparotomy, 18 and 31 %) were required many times more often than for blunt injuries. Lögters et al. reported an emergency thoracotomy rate of 0.5 % and an emergency laparotomy rate of 2.8 % in a total of more than 12,000 patients, almost all of whom had sustained blunt trauma [18]. These results emphasise that the management of penetrating injuries in the resuscitation room requires the presence and expertise of surgeons from different specialties.

This requirement is underlined by the fact that approximately 25 % of all patients with penetrating injuries presented with prehospital haemorrhagic shock (systolic blood pressure ≤ 90 mmHg), which is one of the leading sources of clinical problems. By contrast, less than 10 % of patients with blunt trauma are reported to have a haemorrhagic shock in the prehospital phase [1].

This is not surprising since many other studies found that exsanguation was the leading cause of death in patients with penetrating injuries [12, 19, 20]. Accordingly, patients with penetrating injuries require pRBC transfusions and massive transfusions within the first 48 h considerably more often than patients with blunt trauma. This is the case in approximately 37 % of patients with abdominal injuries [1].

It is undisputed that the primary objective of clinical treatment is to control bleeding into the body cavities, which necessitates surgical intervention in the majority of cases. Permissive hypotension is an approach that is increasingly recommended for the prehospital management of bleeding in body cavities. In the current German S3 Guideline on the Treatment of Patients with severe and multiple injuries, permissive hypotension is a grade B recommendation for the management of actively bleeding patients, which means that this strategy “should” be used, and is contraindicated in patients with injuries to the central nervous system [21]. Recent literature increasingly suggests that permissive hypotension should be rigorously used until surgical control of bleeding has been achieved [22, 23]. Hussmann et al. even reported a survival advantage if this strategy is used [24, 25].

For many years, the TraumaRegister DGU® has reported prehospital rescue times of approximately 70 min for patients with life-threatening injuries [1]. Prehospital rescue times for patients with gunshot or stab wounds are considerably shorter. For example, the rescue time for patients with stab wounds was 58 min and was thus 12 min shorter. Possible reasons may be a lower intubation rate, no need for technical rescue operations, the increased incidence of this injury entity in major cities with a high hospital density, or the use of the “scoop and run” strategy that was intuitively and correctly adopted by the emergency physician.

Penetrating thoracic injuries can lead to acute life-threatening conditions that can be managed by a few simple measures in the prehospital setting. The simplest measure is needle decompression for tension pneumothorax [26]. Several authors recommend that patients with a suspected diagnosis of tension pneumothorax should be managed not only by primary decompression but also by prehospital chest drain insertion. Skin emphysema and serial rib fractures have been suggested as further indications for the prehospital placement of a chest drain in ventilated patients [27]. The current S3 Guideline too does not generally recommend prehospital chest insertion in patients with severe thoracic trauma. According to grade B recommendations, tension pneumothorax should be managed by surgical decompression with or without the placement of a chest drain and pneumothorax should be treated with a chest drain, if indicated [21]. Available data do not sufficiently explain why prehospital chest drain insertion was performed in only 5.6 % of patients with penetrating thoracic trauma (10.1 % of patients with gunshot injuries, 6 % of patients with stab wounds). Likewise, it is unclear to what extent this approach to the patient may have influenced mortality. Further studies should investigate this aspect and should also address the fact that more than 40 % of the chest drains that were placed in the prehospital setting were considered insufficient or inadequate in the resuscitation room. Recently, prehospital clamshell thoracotomy [28] has been repeatedly brought into focus and should be further discussed since this procedure, which should only be performed by an experienced surgeon, causes additional major trauma.

## Limitations

This is a retrospective analysis. A wide variety of factors may have a notable influence and should not be underestimated in the evaluation of findings. Especially the outcome of patients with severe penetrating injuries depends on a multitude of factors (e.g. experience of emergency medical service personnel, time and place of a trauma incident, receiving facility, rescue equipment and vehicles, and patient factors), which, in their entirety, cannot be assessed comprehensively in a register. Furthermore, it should be noted that patients who died in the prehospital setting are not included in the TraumaRegister DGU® (trauma registry of the German Trauma Society). Another limitation of this study is that treatment limitations, for example on the basis of an advance health care directive, were not registered. This applies in particular to the care of patients with severe traumatic brain injury.

It should also be noted that not all German hospitals contribute data to the registry and thus not all patients with life-threatening gunshot and stab wounds were included in this study [1]. Accordingly, the documented and analysed cases on which this study is based are only a sample of patients with gunshot or stab wounds in Germany. Moreover, patients with minor penetrating trauma are not enrolled in the TraumaRegister DGU®. For this reason, the results and conclusions on the overall number of penetrating injuries and especially stab wounds are limited.

## Conclusions

In Germany, gunshot and stab wounds have a low incidence and are mostly caused by violent crime or attempted suicides. Nevertheless, they account for more than half of all penetrating injuries.

Depending on the body region affected, they are associated with a high mortality rate. Moreover, penetrating injuries often lead to a considerable loss of blood that requires early blood transfusion.

Injuries to the chest or abdomen are two-cavity injuries in 50 % of the patients.

Because of the low incidence of these types of penetrating injuries, further data must be collected and analysed with a view to evaluating and improving the quality of long-term care for patients with gunshot and stab wounds. A particular focus should be placed on treatments that provide a survival advantage.

## Data Availability

The datasets during and/or analysed during the current study available from the corresponding author on reasonable request.

## Abbreviations

AIS: Abbreviated Injury Score
ASA: American Society of Anaesthesiologists
CI: confidence interval
d: days
DGU: German Trauma Society
ED: emergency department
e.g.: exempli gratia
fig.: figure
GCS: Glasgow Coma Scale
ICU: intensive care unit
ISS: Injury Severity Score
m: male
pRBC: packed red blood cells
RISC: Revised Injury Severity Classification
SD: Standard deviation
Sektion NIS: Committee on Emergency Medicine, Intensive Care and Trauma Management
SMR: standardised mortality rate
TBI: traumatic brain injury
